# Morphological findings in frozen non-neoplastic kidney tissues of patients with kidney cancer from large-scale multicentric studies on renal cancer

**DOI:** 10.1007/s00428-020-02986-3

**Published:** 2021-01-05

**Authors:** Behnoush Abedi-Ardekani, Dariush Nasrollahzadeh, Lars Egevad, Rosamonde E. Banks, Naveen Vasudev, Ivana Holcatova, Ctibor Povysil, Lenka Foretova, Vladimir Janout, Dana Mates, Viorel Jinga, Amelia Petrescu, Sasa Milosavljevic, Miodrag Ognjanovic, Simona Ognjanovic, Juris Viksna, Anne Y. Warren, Mark Lathrop, Yasser Riazalhosseini, Christine Carreira, Estelle Chanudet, James McKay, Paul Brennan, Ghislaine Scélo

**Affiliations:** 1 International Agency for Research on Cancer, World Health Organization, 150 Cours Albert Thomas, 69372 Lyon Cedex 8, France; 2 Digestive Oncology Research Center, Digestive Diseases Research Institute, Tehran University of Medical Sciences, Tehran, Iran; 3 Department of Oncology-Pathology, Karolinska Institutet, Stockholm, Sweden; 4 Leeds Institute of Medical Research @ St James’s, University of Leeds, Leeds, UK; 5 First Faculty of Medicine, Charles University in Prague, Prague, Czech Republic; 6 Institute of Pathology, First Faculty of Medicine, Charles University and General University Hospital, Prague, Czech Republic; 7 Department of Cancer Epidemiology and Genetics, Masaryk Memorial Cancer Institute, Brno, Czech Republic; 8 Centre of Science and Research, Faculty of Health Sciences, Palacky University, Olomouc, Czech Republic; 9 National Institute of Public Health, Bucharest, Romania; 10 Carol Davila University of Medicine and Pharmacy, Bucharest, Romania; 11 Pathology Department, Professor Dr. Th. Burghele Clinical Hospital, Bucharest, Romania; 12 International Organization for Cancer Prevention and Research, Belgrade, Serbia; 13 Institute of Mathematics and Computer Science, University of Latvia, Riga, Latvia; 14 Cambridge University Hospitals NHS Foundation Trust, Cambridge Biomedical Campus, Cambridge, UK; 15 McGill University and Génome Québec Innovation Centre, Department of Human Genetics, McGill University, Montreal, Quebec Canada

**Keywords:** Kidney cancer, Renal parenchyma, Renal cell carcinoma, Non-neoplastic kidney, Microscopy, Frozen kidney tissue

## Abstract

There are unexplained geographical variations in the incidence of kidney cancer with the high rates reported in Baltic countries, as well as eastern and central Europe. Having access to a large and well-annotated collection of “tumor/non-tumor” pairs of kidney cancer patients from the Czech Republic, Romania, Serbia, UK, and Russia, we aimed to analyze the morphology of non-neoplastic renal tissue in nephrectomy specimens. By applying digital pathology, we performed a microscopic examination of 1012 frozen non-neoplastic kidney tissues from patients with renal cell carcinoma. Four components of renal parenchyma were evaluated and scored for the intensity of interstitial inflammation and fibrosis, tubular atrophy, glomerulosclerosis, and arterial wall thickening, globally called chronic renal parenchymal changes. Moderate or severe changes were observed in 54 (5.3%) of patients with predominance of occurrence in Romania (OR = 2.67, CI 1.07–6.67) and Serbia (OR = 4.37, CI 1.20–15.96) in reference to those from Russia. Further adjustment for comorbidities, tumor characteristics, and stage did not change risk estimates. In multinomial regression model, relative probability of non-glomerular changes was 5.22 times higher for Romania and Serbia compared to Russia. Our findings show that the frequency of chronic renal parenchymal changes, with the predominance of chronic interstitial nephritis pattern, in kidney cancer patients varies by country, significantly more frequent in countries located in central and southeastern Europe where the incidence of kidney cancer has been reported to be moderate to high. The observed association between these pathological features and living in certain geographic areas requires a larger population-based study to confirm this association on a large scale.

**Supplementary Information:**

The online version contains supplementary material available at 10.1007/s00428-020-02986-3.

## Introduction

Worldwide, kidney cancer was reported as the 9th most common malignancy in men and 14th in women in 2018, with an estimated 136,515 and 67,424 new cases in Europe and North America respectively. There is considerable geographic variation in the incidence of kidney cancer; the reason for that is unknown. The highest incidence rates are reported in Baltic countries, as well as Central and Southern Europe, with age-standardized incidence rates of 16.8 in Belarus, followed by Latvia (15.2), Lithuania (14.8), Czech Republic (14.7), and Estonia (14.6) [1]. Renal cell carcinoma (RCC), the most common malignant neoplasm in the kidney, is composed of a heterogeneous group of carcinomas derived from the renal tubular epithelium. The most common carcinomas in this category are clear cell RCC, papillary RCC, and chromophobe RCC, which globally comprise 85–90% of RCC cases.

Previous data indicate that non-neoplastic kidney tissues in nephrectomy specimens, removed for renal neoplasms or advanced renal diseases, are rarely normal and often show variable levels of pathological changes such as glomerular abnormalities, diabetic or hypertensive nephropathies, and chronic pyelonephritis. It is shown that 5 to 25% of RCC patients have varying pathologies of chronic kidney disease and those with normal renal function are more likely to develop chronic kidney disease after nephrectomy. Such changes may cause impairment of renal function and reduce the patient’s quality of life [2–5]. Through examination of non-neoplastic renal parenchymal changes in 110 cancer-related nephrectomy specimens, Bijol et al. showed that only 10% of the cases have unremarkable parenchyma and those with considerable pathological changes such as severe parenchymal scarring and more than 20% glomerulosclerosis had progressive worsening of renal function in 6-month follow-up after radical nephrectomy [6]. Chronic kidney disease and kidney cancer are connected in both directions: the carcinogenic role of chronic kidney diseases has been reported, and conversely, nephrectomy, as the standard therapeutic approach to renal cancer, can lead to chronic kidney disease [7, 8]. Globally, all reports support that while non-neoplastic changes are frequent in nephrectomy specimens, they are often unrecognized, so routine pathological evaluation of non-neoplastic kidney provides valuable diagnostic and prognostic information. On the other hand, understanding molecular events such as somatic mutations in non-neoplastic cells is central to understand cancer development and is taking a considerable place in research on cancer [9, 10].

During 2007 to 2016, the International Agency for Research on Cancer (IARC)/WHO conducted hospital- and population-based, multicentric case-control studies in the Czech Republic, Romania, Serbia, and Russia (K2 study) to recruit incident cases with a kidney cancer diagnosis for the purpose of a better understanding of the epidemiology, etiology, and genomics of renal cancer. In parallel, incident kidney cancer cases were recruited in the United Kingdom (UK) by the Leeds Multidisciplinary Research Tissue Bank. Both collections were used in the Cancer Genomic of Kidney (CAGEKID) project (http://www.cng.fr/cagekid/). This collection comprises more than 2000 frozen “tumor/non-tumor” pairs with annotated clinico-epidemiological data of kidney cancer patients.

Having access to this multicentric and international collection, we designed this study to evaluate the morphology of post-nephrectomy non-neoplastic kidney tissues in a subset of the patients with RCC from our collection.

## Material and methods

### Study design

Collection of renal tissue pairs in both K2 and CAGEKID studies was performed by applying harmonized standard operative procedures (SOPs) throughout the participating countries. Immediately after partial or radical nephrectomy, in addition to tumor tissue, one piece of non-neoplastic tissue was collected with the following specifications: from the renal cortex, distant from tumor, and at least 5 mm in the largest diameter. However, the exact distance of the collected non-tumor tissue from tumor was not recorded. All of the collected tissues were snap-frozen and stored in liquid nitrogen or directly immersed in RNA*later* followed by freezing in − 80 °C freezers and were then shipped to IARC for storage and processing. Patients were interviewed and detailed information including tobacco use, alcohol consumption, body mass, medical history, and family history of cancer, as well as clinical data from medical records, were collected. Digital pathology was applied for microscopic examination of frozen tissues by a panel of expert pathologists. Non-tumor tissues were examined by a single pathologist, mainly to confirm the absence of tumor contamination, to be used in gene expression analyses.

The current study took shape accidentally and following some specific observations. We at IARC, responsible for DNA/RNA extraction for the CAGEKID study, noticed lower RNA quality in non-tumor tissues compared to tumors. While looking for the reason behind this observation and finding none, we decided to have a second look at the microscopy of non-tumor tissues to understand if there could be any morphological finding relevant to this observation. BAA, pathologist at IARC, performed this examination and evaluated and recorded the observed features, as detailed as possible. The pathologist was blinded to tumor type, epidemiological data, country of residence, and results of RNA quality. This first essay on 626 tissues ended to find nothing that could explain the quality of RNA. Instead, we found some pathologies that our preliminary statistical analyses showed them occurring significantly limited to specific countries. These unexpected but interesting results led us to expand our work to a larger number of available tissues in our biorepository and to re-evaluate them altogether under one project and by applying more rigorous statistical analyses. Below, we report the design and the results of this work.

We selected 1045 non-neoplastic kidney tissues based on the availability of at least one hematoxylin and eosin (H&E) stained slide, irrespective of the tumor type or the country of recruitment. High-resolution whole slide images were used for digital microscopy by one pathologist (BAA), blind to the type and status of tumor, demographic and clinical data, country of recruitment, or laboratory results. We first evaluated the composition of renal parenchyma, and if purely from cortex or containing medulla as well. Four components of renal cortex and their pathological changes were examined: renal glomeruli and the presence of various degrees of glomerular sclerosis (GS); renal tubules and the presence and severity of atrophy; presence and severity of interstitial fibrosis (the latter two together called IFTA); presence, severity, and type of inflammation; and also blood vessels’ structure. All the observed changes were recorded. We then qualitatively scored the intensity of these changes, which were collectively called chronic renal parenchymal changes (CRPC), from none to severe as follows: none, if no significant pathological changes were observed, and mild, moderate, and severe if any or a complex of parenchymal changes were seen with +, ++, or +++ intensity, respectively. We then used the proposed scoring method by the International Society of Nephrology (ISN) to report the microscopic findings of the non-neoplastic renal tissues in native kidney biopsies. In ISN scoring, the severity of changes in each renal parenchymal component is scored quantitatively, and by adding the scores the severity of chronic changes are graded into minimal, mild, moderate, and severe [11]. By applying ISN scoring as the gold standard, we re-examined and newly scored all available images that we initially scored as having mild, moderate, or severe CRPC.

### Statistical analysis

Dependent on our findings, we categorized outcomes into two main groups: those with none–mild CRPC versus those with moderate–severe CRPC. Demographic variables were then categorized and compared between two outcomes including country of recruitment, age at diagnosis, body mass index (BMI) at diagnosis, tobacco smoking status, and alcohol consumption habit. Furthermore, we added variables that could have a negative impact on our collection protocol such as tumor size and stage, surgical approach (radical or partial), and percentage of the medulla (the predominance of medulla was considered as a possible indicator of full occupation of cortex by tumor). Kidney-related diseases or medical conditions that could potentially confound the relation between CRPC and demographic variables were analyzed, including self-reported history of hypertension, diabetes mellitus, nephrolithiasis, chronic kidney disease, and ever use of non-steroidal anti-inflammatory drugs (NSAIDs). Logistic regression models were used to calculate unadjusted and adjusted odds ratios (ORs) and 95% confidence intervals (CIs). In all analyses, unexposed groups were the reference. For numerical variables, lowest category was considered as a reference. To study the association between country and CRPC, we used three models to include co-variables for adjustment: model 1 includes age (5 levels of age groups), sex, and percentage of the medulla (4 categories); in model 2, we added stage of the tumor (4 levels) and tumor size ( 4 categories); in model 3, we added 3 binary variables including history of diabetes mellitus, hypertension, and NSAID use to model 1.

We evaluated the performance of our scoring by comparing it with the ISN scoring method. Also, we used multinominal regression analysis to compare geographical residence (as predictor) association with the presence of individual components of CRPC without applying any score which included: only GS and at any level, pathologies limited to IFTA with no GS, presence of both GS and IFTA, and absence of any pathological change. Age, gender, and presence of inflammation were included in the model.

## Results

The examined images had the good quality for the purpose of this evaluation (Supplementary Figures 1 to 7). Of the 1045 initially selected tissues, 33 were excluded due to the exclusive presence of renal medulla in which evaluation of renal cortical structures was not possible. Table 1 summarizes the demographic characteristics as well as potential confounders of the examined patients by grouping them into two categories of CRPC as the outcome of interest. In 1012 examined ones, 836 (82.6%) did not show significant parenchymal changes (Supplementary Figure 1), 122 (12.0%) were scored as mild CRPC (Supplementary Figure 2), 28 (2.8%) as moderate (Supplementary Figure 3 and 4), and 26 (2.6%) as severe CRPC (Supplementary Figures 5 to 7). 958 (94.7%) were grouped into none–mild CRPC category, and 54 (5.3%) subjects met our criteria for being considered as having moderate–severe CRPC. Except for age and country of recruitment, no significant difference was observed between the proportion of moderate–severe CRPC relative to tumor type, sex, BMI, smoking habit, and alcohol consumption. The mean age of patients recruited from Russia and the UK was 57 and 62 years old. Patients from Serbia, Romania, and the Czech Republic were recruited at the mean age of 60, 63, and 64 years. In univariate analysis, the odds of having moderate–severe CRPC showed a significant trend with older age (*P* = 0.001). The proportion of both glomerular and tubular components of CRPC increased by age (Supplementary Figure 8).

**Table 1:** Demographic characteristics of patients with moderate–severe chronic renal parenchymal changes and otherwise in non-neoplastic kidney tissues

	Chronic renal parenchymal changes		*P* value
	None–mild	Moderate–severe	
	*N* = 958	*N* = 54	
Recruiting country			
Czech Republic	402 (42.0)	24 (44.4)	0.007
Romania	271 (28.3)	9 (16.7)	
UK	144 (15.0)	4 (7.4)	
Russia	115 (12.0)	13 (24.1)	
Serbia	26 (2.7)	4 (7.4)	
Sex			
Male	601 (62.7)	32 (59.3)	0.6
Female	357 (37.3)	22 (40.7)	
Age at diagnosis			
< 55	238 (24.8)	6 (11.1)	0.02
55–59	162 (16.9)	5 (9.3)	
60–64	190 (19.8)	11 (20.4)	
65–69	156 (16.3)	13 (24.1)	
> 70	212 (22.1)	19 (35.2)	
BMI at diagnosis			
< 24.9	221 (23.1)	14 (25.9)	0.9
25.0–29.9	364 (38.0)	19 (35.2)	
30.0–34.9	224 (23.4)	14 (25.9)	
> 35.0	88 (9.2)	6 (11.1)	
Missing	61 (6.4)	1 (1.8)	
Tobacco smoking status			
Never smoker	484 (50.5)	24 (44.4)	0.4
Ex-smoker	232 (24.2)	18 (33.3)	
Current smoker	233 (24.3)	12 (22.2)	
Missing	9 (0.9)	0 (0.0)	
Alcohol drinking status			
Never drinker	481 (50.2)	29 (53.7)	0.8
Ex-drinker	86 (9.0)	7 (13.0)	
Current drinker	241 (25.2)	14 (25.9)	
Missing	150 (15.6)	4 (7.4)	

There was a variation in the number of recruited cases in this study. The Czech Republic with 426 patients provided the highest number of cases from four different recruiting cities, followed by Romania (Bucharest), the UK (Leeds), Russia (Moscow), and Serbia (Belgrade). Clear cell RCC was the most common histological type of RCC in the entire examined patients (84.0%), followed by papillary RCC (7.3%). Other tumor types included chromophobe RCC, collecting duct carcinoma, unclassified RCC, and oncocytoma, which altogether comprised 8.7% of the total examined cases. The proportion of major RCC histological types did not vary across CRPC scores (Table 2). The relative probability of detecting GS rather than no changes for patients from the Czech Republic is more than double the corresponding probability for patients from Russia or the UK with the same level of inflammation. Variable degrees of GS were not significantly higher among Romanians and Serbians compared to Russians. In contrast, IFTA was observed four and five times higher in patients from the Czech Republic and Romania-Serbia, respectively, than Russians. The relative probability remained unchanged when those with both GS and IFTA were tested (Table 3).

**Table 2:** Comparison of severity of chronic renal parenchymal changes in non-neoplastic kidney tissues with tumor type and recruitment country

	None–mild						Moderate–severe					
RCC type	Czech	Romania	Russia	Serbia	UK	Total	Czech	Romania	Russia	Serbia	UK	Total
Clear cell	321	231	93	18	144	807	19	8	10	2	4	43
Papillary	42	16	4	6	0	68	3	1	1	1	0	6
Other	39	24	18	2	0	83	2	0	2	1	0	5

**Table 3:** Odds ratio of observing four groups of parenchymal changes relevance to geographic residence of cancer patients (adjusted for age, gender, and presence of inflammation)

Study region	None (reference)	Only glomerulosclerosis (GS)		Interstitial fibrosis or tubular atrophy (IFTA)		Both GS and IFTA	
	*N* (%)	*N* (%)	OR (95%CI)	*N* (%)	OR (95%CI)	*N* (%)	OR (95%CI)
Russia	255 (31.7)	15 (15.8)	Reference	3 (8.6)	Reference	6 (7.7)	Reference
Czech Republic	303 (37.7)	56 (58.9)	2.41* (1.22–4.74)	22 (62.9)	4.38* (1.20–15.93)	45 (57.7)	4.29** (1.53–12.04)
Romania and Serbia	110 (13.7)	18 (18.9)	2.16 (0.95–4.88)	10 (28.6)	5.22* (1.30–20.98)	21 (26.9)	5.51** (1.77–17.10)
UK	136 (16.9)	6 (6.3)	0.50 (0.17–1.48)	0 (0)	NA	6 (7.7)	1.03 (0.25–4.191)

*p < 0.05

**p < 0.01

Table 4 summarizes the result of our analysis to evaluate whether the observed morphological changes were secondary to the effect of the tumor on adjacent tissue or self-reported medical conditions. We used variables such as presence and percentage of the medulla in non-neoplastic tissues, size of the tumor, stage at which tumor was operated, and type of surgical approach to study their impact on the outcome. Radical nephrectomy was the surgical method of choice in 86% of Russians, 81% of the Czech Republic, and 98% of Romanian-Serbian cases. The proportion of tumors larger than 10 cm was similar among Russian and Romanian-Serbian patients (12.5%) and over twice among Czech Republic cases (25%). Advanced tumor stage (III and IV) was observed in 27%, 42%, and 38% of patients from Russia, the Czech Republic, and Romania-Serbia groups. Our results indicate that apart from surgeon preference, tumor size was not the major drive for higher proportion of radical nephrectomies in the group of Romania-Serbia. Furthermore, we observed a similar proportion of radical nephrectomy between cases with CRPC (88.9%) and non-CRPC (87.1%). We compared the method of choice for surgical resection between GS and IFTA components of CRPC and we observed that among those with predominant GS, 13% underwent partial nephrectomy and 17.8% were operated by radical nephrectomy approach (*P* = 0.20). Among cases with predominant IFTA, surgical approaches were 7.8% and 8.8% for partial and radical nephrectomy (*P* = 0.72).

**Table 4:** Tumor and medical variables among two categories of patients subdivided by presence/severity of chronic renal parenchymal changes in non-neoplastic kidney tissues

	Chronic renal parenchymal changes		*P* value
	None–mild	Moderate–severe	
	*N* = 958	*N* = 54	
Presence of medulla (%)			
0–9	687 (71.7)	41 (71.9)	0.9
10–30	154 (16.1)	8 (14.8)	
31–50	51 (5.3)	3 (5.6)	
> 50	66 (6.9)	2 (3.7)	
Type of nephrectomy			
Radical	834 (87.1)	48 (88.9)	0.6
Partial	109 (11.4)	6 (11.1)	
Unknown/missing	15 (1.6)	0 (0.0)	
Stage at diagnosis			
I	430 (44.9)	18 (33.3)	0.6
II	99 (10.3)	7 (13.0)	
III	190 (19.8)	12 (22.2)	
IV	127 (13.3)	9 (16.7)	
Missing	112 (11.7)	8 (14.8)	
Tumor size (cm)			
*T* **≤** 4	189 (19.7)	7 (13.0)	0.4
4 < *T* **≤** 7	373 (38.9)	26 (48.0)	
7 < *T* **≤** 10	221 (23.1)	11 (20.4)	
*T* > 10	164 (17.1)	8(14.8)	
Missing	11 (1.0)	2 (3.7)	
History of hypertension			
No	466 (48.6)	23 (42.6)	0.4
Yes	480 (50.1)	31 (57.4)	
Missing	12 (1.2)	0 (0.0)	
History of diabetes			
No	700 (73.1)	38 (70.4)	0.04
Yes	109 (11.4)	12 (22.2)	
Missing	149 (15. 5)	4 (7.4)	
History of chronic renal disease			
No	728 (76.0)	44 (81.5)	0.2
Yes	26 (2.7)	3 (5.6)	
Missing	204 (21.3)	7 (13.0)	
Regular NSAID user			
No	676 (70.6)	37 (68.5)	0.02
Yes	123 (12.8)	13 (24.1)	
Missing	159 (16.6)	4 (7.4)	

Since medical conditions such as hypertension and diabetes mellitus are common in RCC patients and could lead to morphological changes in renal parenchyma, we evaluated the association of the observed CRPC with medical conditions and risk factors for RCC. Almost half of RCC patients reported history of hypertension in this study. In spite of variation in prevalence of hypertension across countries, hypertensive patients were equally distributed among CRPC categories (*P* = 0.4). Self-reporting of diabetes mellitus was recorded in 12.0% of patients. Similar proportion of our two CRPC groups did not report history of diabetes (73.0% vs. 70.0%). Although percentage of missing data for diabetes was significantly higher in none–mild category, there was a trend towards less cases with diabetes among moderate–severe CRPC (*P* = 0.03). In our series, 121 patients reported a history of diabetes (18% of Russians, 10% of Czechs, 11% of Romanians-Serbians). A total of 511 patients had a history of hypertension (64% of Russians, 41% of Czechs, and 48% of Romanians-Serbians). NSAIDs users were defined as those who take NSAIDs at least once a week for a year. Both groups of CRPC showed a similar proportion of non-users (68.5% vs. 70.6%) while missing data amongst advanced CRPCs was less than half of none–mild group (7.0% vs. 17.0%). After excluding missing data, NSAIDs use was less common among moderate–severe CRPC group than none–mild one (*P* = 0.04).

Table 5 shows ORs for association between country of recruitment and the CRPCs as outcome. Due to limited number of events in Serbia (with modest sample size), we pooled Serbia and Romania to achieve more stable estimate. Minimally adjusted model (including age, gender, and the percentage of medulla) showed that those living in Romania and Serbia experienced more than threefold increased risk of moderate–severe CRPC comparing to those living in Russia or the UK. Neighboring geographic sites (Czech Republic) shows a 1.7-fold increase in risk, although this point estimate does not remain significant. Adjustment for tumor size, stage, history of hypertension, history of diabetes, and regular NSAIDs use did not materially change the estimate.

**Table 5:** Odds ratio and 95% confidence interval for association between country and observing moderate–severe chronic renal parenchymal changes in non-neoplastic kidney tissues of RCC patients

Recruiting country	Odds ratio (95% CI)			
	Unadjusted	Model 1*	Model 2**	Model 3***
Russia	Reference	Reference	Reference	Reference
UK	0. 84 (0.25–2.76)	0.64 (0.19–2.16)	0.59 (0.17–2.01)	0.39 (0.10–1.49)
Czech Republic	1.79 (0.82–3.92)	1.34 (0.59–3.00)	1.54 (0.67–3.57)	1.28 (0.56–2.91)
Romania	*3.40 (1.41–8.18)*	*3.12 (1.26–7.74)*	*3.16 (1.24–8.03)*	*2.67 (1.07–6.67)*
Serbia	*4.63 (1.33–16.08)*	*5.06 (1.39–18.44)*	*6.27 (1.40–28.02)*	*4.37 (1.20–15.96)*
Romania and Serbia	*3.62 (1.57–8.32)*	*3.42 (1.44–8.12)*	*3.53 (1.45–8.58)*	*2.96 (1.24–7.03)*

*Adjusted for age, gender, percentage of medulla

**Adjusted for age, gender, percentage of medulla, stage, tumor size

***Adjusted for age, gender, diabetes, hypertension, NSAID use

Comparing our scoring method and the ISN showed that the two approaches are highly correlated. Estimated diagnostic accuracy of grading used in this study with the gold standard was calculated based on 82% sensitivity (95% CI: 59.7%–94.8%), 100% specificity (95% CI: 87.2%–100%), and 0.91 area under ROC (95% CI: 0.83–0.99) (Supplementary Table 1).

## Discussion

Here, we explain the morphological features of renal parenchyma in frozen non-neoplastic tissues of patients with RCC in different geographical sites across central and eastern Europe, Russia, and the UK, and report a threefold increase in odds of having CRPC associated with living in Romania and Serbia. These findings are mainly reported by one pathologist (BAA) who was also member of the expert pathology panel of the CAGEKID study. We acknowledge the importance of long-time expertise in uro-nephro pathology to examine renal tissues, although our findings show that the level of expertise might not differentially affect the classification of tissues into CRPC and otherwise between countries and it is unlikely that the observed difference is due to this aspect of study design.

Previous reports on the microscopic examination of non-neoplastic kidney tissue in cancer-related nephrectomies have shown that the pressure effect from the adjacent tumor causes pathologic morphological and clinical features while this effect reduces with distance. Azhar et al. showed that the severity of pathological findings such as inflammation, nephrosclerosis, and glomerulosclerosis decreased from severe to mild at 1 to 5 mm distance from tumor [12]. Studies focused on functionality of adjacent-tumor non-neoplastic renal parenchyma shows glomerular viability reaching to 92% in 1.0 cm from tumor [13].

To address the possibility of tumor pressure effect being the origin of our observations, we first relied on our harmonized sample collection procedure in which collection of non-neoplastic tissues as distant as possible from tumor, and from renal cortex has been emphasized. Based on these guidelines, we expect that these tissues were collected distant from tumor, particularly in more than 5 mm (the distance limit where the severity of pathological changes caused by tumor decreases considerably). The observed association between CRPC and country of origin could not be entirely explained by possibility of collecting nontumoral tissues closer to tumor in Romania and Serbia, notably because we observed lower number of tumors larger than 10 cm in these countries. Also, choice of surgical method and tumor stage proportion of cases with GS or IFTA was similar between patients with and without CRPC. In addition, all of the examined non-neoplastic tissues were free of tumor in microscopic examination. Adjusted models for confounders such as tumor size and type of nephrectomy did not change the initial risk estimates. These findings support the idea that the observed CRPC are not caused by the pressure effect from tumor and are independent events.

Hypertension and diabetes mellitus are among the main causes of chronic and end-stage renal disease and lead to pathological parenchymal features mainly in arterioles and glomerular capillaries. For precise evaluation of these changes, special stains such as periodic acid-Schiff and methenamine silver should complete the H&E staining. Due to the frozen nature of our samples, we could not perform special stains. Instead, we applied strict criteria to overcome this limitation. We categorized all of the mild changes with those with no significant pathological changes, in the group of none–mild to avoid overestimation of the observations and to cover any possibility of missing some diagnostic features due to the use of frozen tissues. Our qualitative scoring was not only correlated with the quantitative one by ISN but also lower sensitivity in this method ensures us that our estimated risk might be even underestimated due to misclassification of true positive ones among CRPC negative group. In addition, lack of hypertension and diabetes mellitus history was similarly reported in the both groups of none–mild and moderate–severe CRPC. When adjusted for these factors, the odds estimate did not change. Self-reported hypertension and diabetes mellitus were less frequent among Romanians. As a result, these two common medical conditions are more frequent in our reference population (Russians) and cannot fully explain the higher prevalence of CRPC in patients from the Czech Republic or Romania and Serbia.

The significant presence of CRPC in Romania and Serbia is of particular interest. Based on the 2019 Global Burden of Disease (GBD) study database (http://www.healthdata.org/gbd), and WHO data observatory (https://www.who.int/gho/en/), the prevalence of chronic kidney disease (CKD) in the Czech Republic, Romania, and Serbia is 13.5%, 12.3%, and 13.3% respectively. In our study, the report of medical history of CKD in the Czech Republic and Romania with 426 and 128 recruited cases was 3.5% and 3.9%, respectively. The prevalence of CKD in our study group from these two countries is lower than the reported country prevalence with no statistical difference between two countries. Among 31 Serbian cases, only 6 reported a history of CKD. Due to the modest sample size, we combined patients from Serbia and Romania. CKD prevalence in the combined group reaches to 6.9%. There is no statistical difference between CKD prevalence in the Czech Republic versus combined Romania and Serbia (Pearson’s chi-squared = 2.99, *P* = 0.08).

We showed that IFTA is the main driver for the observed difference between the occurrence and severity of CRPC across study regions. Interstitial fibrosis and inflammation, with tubular atrophy, were the predominant features and glomerular and vascular changes seem to stem from the interstitial disease. These features are more suggestive for a chronic interstitial nephritis (CIN) pattern. CIN is a nonspecific diagnosis and identification of a specific reason causing it is not based on light microscopy only and needs accurate clinical history and imaging studies, the elements that are missing in our study. Chronic pyelonephritis, analgesic nephropathy, and Balkan endemic nephropathy (BEN) are among the most important specific reasons leading to CIN. BEN was first reported in the 1950s as a progressive renal disease in the form of chronic interstitial nephritis affecting the people living in the Balkan Peninsula along the tributaries of the Danube River in Bosnia and Herzegovina, Bulgaria, Croatia, Romania, and Serbia. Data support the etiologic role of dietary exposure to aristolochic acid (AA) in both BEN and Chinese herbs nephropathy. The association of AA nephropathy with urothelial carcinoma of the upper urinary tract is shown but such a link with RCC is not reported [14–16]. We have previously published the results of whole-genome sequencing of 94 RCC patients from the CAGEKID that revealed a striking elevation of A:T.T:A transversions in 12 out of 14 RCC patients from Romania, a mutational pattern that is consistent with the exposure to AA [17]. Although the microscopic examination of non-neoplastic tissues of those patients did not show typical characteristics of BEN, variably high levels of AA DNA adducts, ranging from 0.7 to 27.0 adducts per 10^8^, were detected in them [18]. Eight of those 14 patients were included in this study. Similar to the previous report, we did not find any characteristic morphological feature indicative of BEN. Only two patients had focal and mild interstitial fibrosis and inflammation, categorized in none–mild group, but with AA DNA adduct level reported as 26.84 and 6.08 per 10^8^ DNA bases. To further investigate the mystery of RCC in these countries, a large collection of kidney cancer patients from the Czech Republic, Romania, and Serbia will be soon analyzed for the discovery of mutational signatures caused by environmental exposures as part of our ongoing collaborative project, Mutographs of cancers (https://www.mutographs.org) that might lead to answer some of the questions in this regard.

To our best knowledge, this study is unique and the first one to report pathological changes in non-neoplastic kidney tissues regarding both sample size and geographical diversity of the patients from countries with variable incidence of RCC. That is also the first report from Serbia where we observed the highest proportion (13.3%) of moderate–severe changes in renal parenchyma.

Additionally, this is the first report relying on frozen tissues for detailed microscopic examination. In the current era of a growing number of molecular studies based on the use of frozen tissues to optimize the quality of the high throughput technologies, it is critical to assess the reliability of morphological examination of frozen tissues. It was worth mentioning that properly preserved frozen tissues have acceptable quality for microscopic examination, although one should be aware of their limitations. At IARC, we are experienced in treating and evaluating variable kinds of frozen tissues and performing the routine microscopic evaluation of frozen tissues for the purpose of genomic studies.

Our study has some limitations. Undoubtedly, we could have better verified our findings on formalin-fixed paraffin-embedded (FFPE) tissues that are the gold standard in diagnostic pathology. However, we did not have access to FFPE blocks because of the retrospective nature of the study, collection of samples in more than 10 years ago, and multicentric nature of the collection that complicates the access to the pathology archives of the hospitals. The main limitation of use of frozen tissues in this study is inability to apply special stains, which are part of routine practice of nephropathology, particularly to evaluate glomerular and vascular changes. However, H&E is good enough for the microscopic evaluation of interstitial and tubular changes, the predominant observed pathological features.

Additionally, our study suffers from missing clinical and laboratory data of renal function, because it is based on retrospective collection that was designed for another aim. However, our findings bring the first level of information and urge for properly designed studies to answer the raised questions.

In conclusion, we report renal parenchymal pathologies in non-neoplastic kidney tissue of RCC patients in central and southeastern Europe with the predominance of CIN pattern rather than primary glomerular disease. We show that these findings are unlikely to be due to sampling method or pressure effect of the tumor alone. The observed association between CRPC and living in certain geographic areas requires larger population-based studies to confirm this association in large-scale and designed studies for this aim.

## Supplementary information

ESM 1 (PPTX 7.17 mb)

ESM 2 (DOCX 15.1 kb)

## Data Availability

The datasets generated during this study are not publicly available due to the presence of confidential participants’ information but are available from the corresponding author upon reasonable request.

## References

[1] Ferlay J, Colombet M, Soerjomataram I, Mathers C, Parkin DM, Pineros M, Znaor A, Bray F (2019). Estimating the global cancer incidence and mortality in 2018: GLOBOCAN sources and methods. Int J Cancer.

[2] Wee JW, Kang HR, Kwon SH, Jeon JS, Han DC, Jin SY, Yang WJ, Noh H (2016). Clinical value of pathologic examination of non-neoplastic kidney in patients with upper urinary tract malignancies. Korean J Intern Med.

[3] Truong LD, Shen SS, Park MH, Krishnan B (2009). Diagnosing nonneoplastic lesions in nephrectomy specimens. Arch Pathol Lab Med.

[4] Henriksen KJ, Meehan SM, Chang A (2009). Nonneoplastic kidney diseases in adult tumor nephrectomy and nephroureterectomy specimens: common, harmful, yet underappreciated. Arch Pathol Lab Med.

[5] Sarsik B, Simsir A, Yilmaz M, Yorukoglu K, Sen S (2013). Spectrum of nontumoral renal pathologies in tumor nephrectomies: nontumoral renal parenchyma changes. Ann Diagn Pathol.

[6] Bijol V, Mendez GP, Hurwitz S, Rennke HG, Nose V (2006). Evaluation of the nonneoplastic pathology in tumor nephrectomy specimens: predicting the risk of progressive renal failure. Am J Surg Pathol.

[7] Stengel B (2010). Chronic kidney disease and cancer: a troubling connection. J Nephrol.

[8] Chang A, Finelli A, Berns JS, Rosner M (2014). Chronic kidney disease in patients with renal cell carcinoma. Adv Chronic Kidney Dis.

[9] Martincorena I, Roshan A, Gerstung M, Ellis P, Van Loo P, McLaren S, Wedge DC, Fullam A, Alexandrov LB, Tubio JM, Stebbings L, Menzies A, Widaa S, Stratton MR, Jones PH, Campbell PJ (2015). Tumor evolution. High burden and pervasive positive selection of somatic mutations in normal human skin. Science.

[10] Yoshida K, Gowers KHC, Lee-Six H, Chandrasekharan DP, Coorens T, Maughan EF, Beal K, Menzies A, Millar FR, Anderson E, Clarke SE, Pennycuick A, Thakrar RM, Butler CR, Kakiuchi N, Hirano T, Hynds RE, Stratton MR, Martincorena I, Janes SM, Campbell PJ (2020). Tobacco smoking and somatic mutations in human bronchial epithelium. Nature.

[11] Sethi S, D’Agati VD, Nast CC, Fogo AB, De Vriese AS, Markowitz GS, Glassock RJ, Fervenza FC, Seshan SV, Rule A, Racusen LC, Radhakrishnan J, Winearls CG, Appel GB, Bajema IM, Chang A, Colvin RB, Cook HT, Hariharan S, Herrera Hernandez LP, Kambham N, Mengel M, Nath KA, Rennke HG, Ronco P, Rovin BH, Haas M (2017). A proposal for standardized grading of chronic changes in native kidney biopsy specimens. Kidney Int.

[12] Azhar RA, de Castro Abreu AL, Broxham E, Sherrod A, Ma Y, Cai J, Gill TS, Desai M, Gill IS (2015). Histological analysis of the kidney tumor-parenchyma interface. J Urol.

[13] Khemees TA, Lam ET, Joehlin-Price AS, Mortazavi A, Phillips GS, Shabsigh A, Sharp DS, Zynger DL (2016). Does the renal parenchyma adjacent to the tumor contribute to kidney function? A critical analysis of glomerular viability in partial nephrectomy specimens. Urology.

[14] Stefanovic V, Polenakovic M (2009). Fifty years of research in Balkan endemic nephropathy: where are we now?. Nephron Clin Pract.

[15] Stefanovic V, Polenakovic M, Toncheva D (2011). Urothelial carcinoma associated with Balkan endemic nephropathy. A worldwide disease. Pathol Biol (Paris).

[16] Pavlovic NM (2013). Balkan endemic nephropathy-current status and future perspectives. Clin Kidney J.

[17] Scelo G, Riazalhosseini Y, Greger L, Letourneau L, Gonzalez-Porta M, Wozniak MB, Bourgey M, Harnden P, Egevad L, Jackson SM, Karimzadeh M, Arseneault M, Lepage P, How-Kit A, Daunay A, Renault V, Blanche H, Tubacher E, Sehmoun J, Viksna J, Celms E, Opmanis M, Zarins A, Vasudev NS, Seywright M, Abedi-Ardekani B, Carreira C, Selby PJ, Cartledge JJ, Byrnes G, Zavadil J, Su J, Holcatova I, Brisuda A, Zaridze D, Moukeria A, Foretova L, Navratilova M, Mates D, Jinga V, Artemov A, Nedoluzhko A, Mazur A, Rastorguev S, Boulygina E, Heath S, Gut M, Bihoreau MT, Lechner D, Foglio M, Gut IG, Skryabin K, Prokhortchouk E, Cambon-Thomsen A, Rung J, Bourque G, Brennan P, Tost J, Banks RE, Brazma A, Lathrop GM (2014). Variation in genomic landscape of clear cell renal cell carcinoma across Europe. Nat Commun.

[18] Turesky RJ, Yun BH, Brennan P, Mates D, Jinga V, Harnden P, Banks RE, Blanche H, Bihoreau MT, Chopard P, Letourneau L, Lathrop GM, Scelo G (2016). Aristolochic acid exposure in Romania and implications for renal cell carcinoma. Br J Cancer.

