# Supplementary material for: Morphological findings in frozen non-neoplastic kidney tissues of patients with kidney cancer from large-scale multicentric studies on renal cancer
Source: Virchows Arch. 2021 Jan 5;478(6):1099–107. doi: 10.1007/s00428-020-02986-3 (PMC8203524; doi:10.1007/s00428-020-02986-3)
Supplement: Supplementary file 1 — (PPTX 7.17 mb) [file 428_2020_2986_MOESM1_ESM.pptx]

## Slide 1
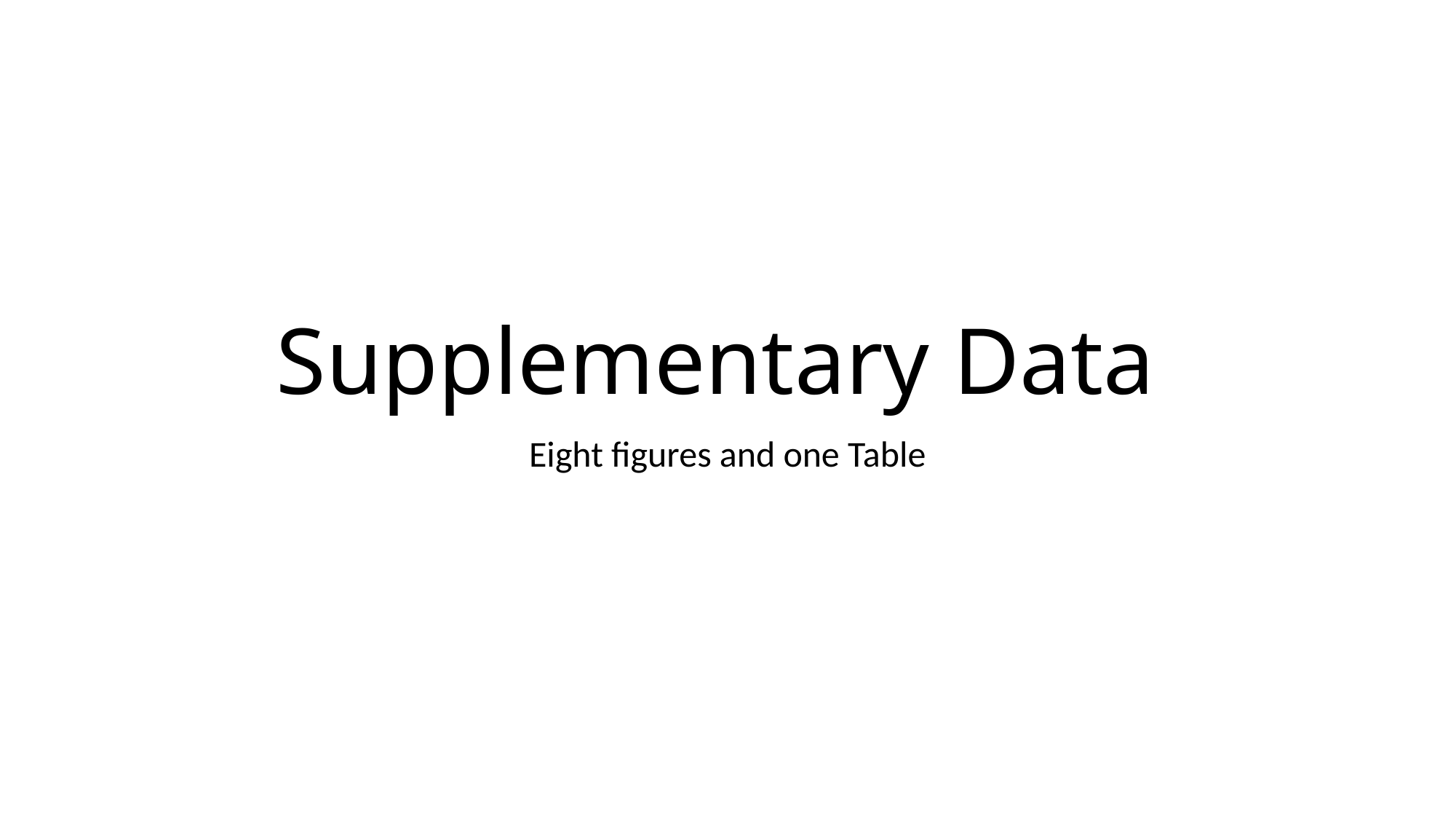

# Supplementary Data
Eight figures and one Table

## Slide 2
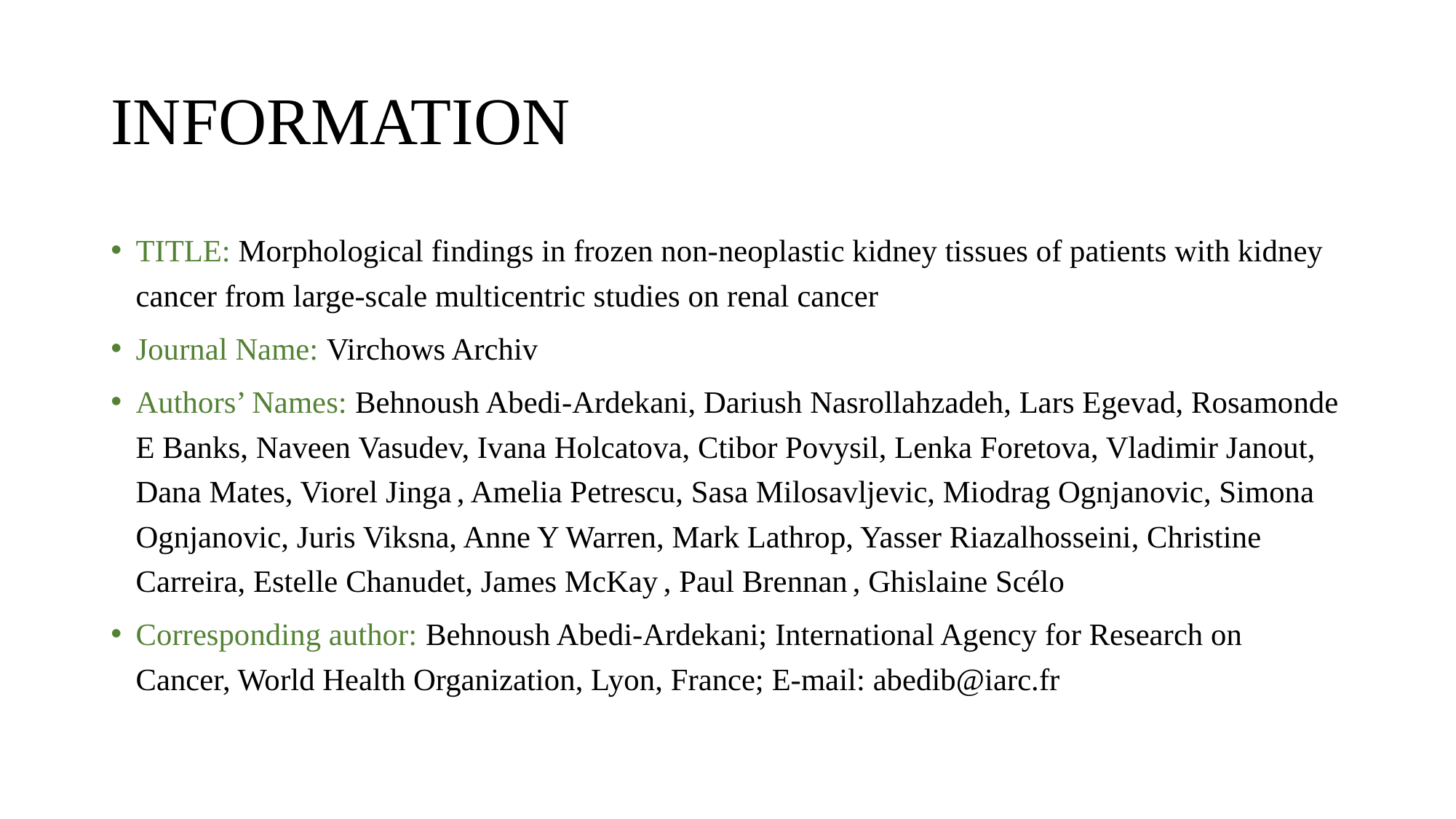

# INFORMATION
TITLE: Morphological findings in frozen non-neoplastic kidney tissues of patients with kidney cancer from large-scale multicentric studies on renal cancer
Journal Name: Virchows Archiv
Authors’ Names: Behnoush Abedi-Ardekani, Dariush Nasrollahzadeh, Lars Egevad, Rosamonde E Banks, Naveen Vasudev, Ivana Holcatova, Ctibor Povysil, Lenka Foretova, Vladimir Janout, Dana Mates, Viorel Jinga , Amelia Petrescu, Sasa Milosavljevic, Miodrag Ognjanovic, Simona Ognjanovic, Juris Viksna, Anne Y Warren, Mark Lathrop, Yasser Riazalhosseini, Christine Carreira, Estelle Chanudet, James McKay , Paul Brennan , Ghislaine Scélo
Corresponding author: Behnoush Abedi-Ardekani; International Agency for Research on Cancer, World Health Organization, Lyon, France; E-mail: abedib@iarc.fr

## Slide 3
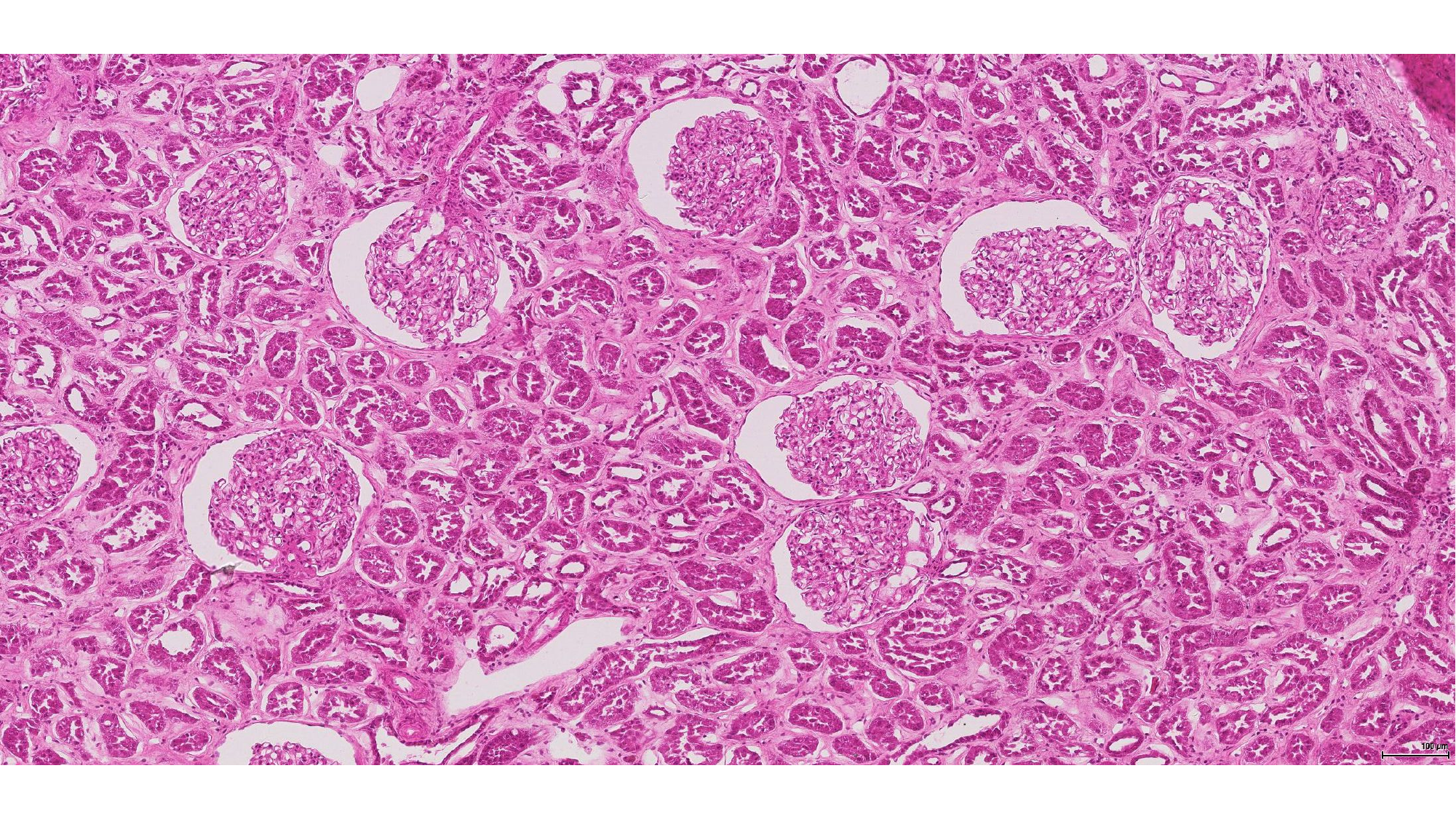

## Slide 4
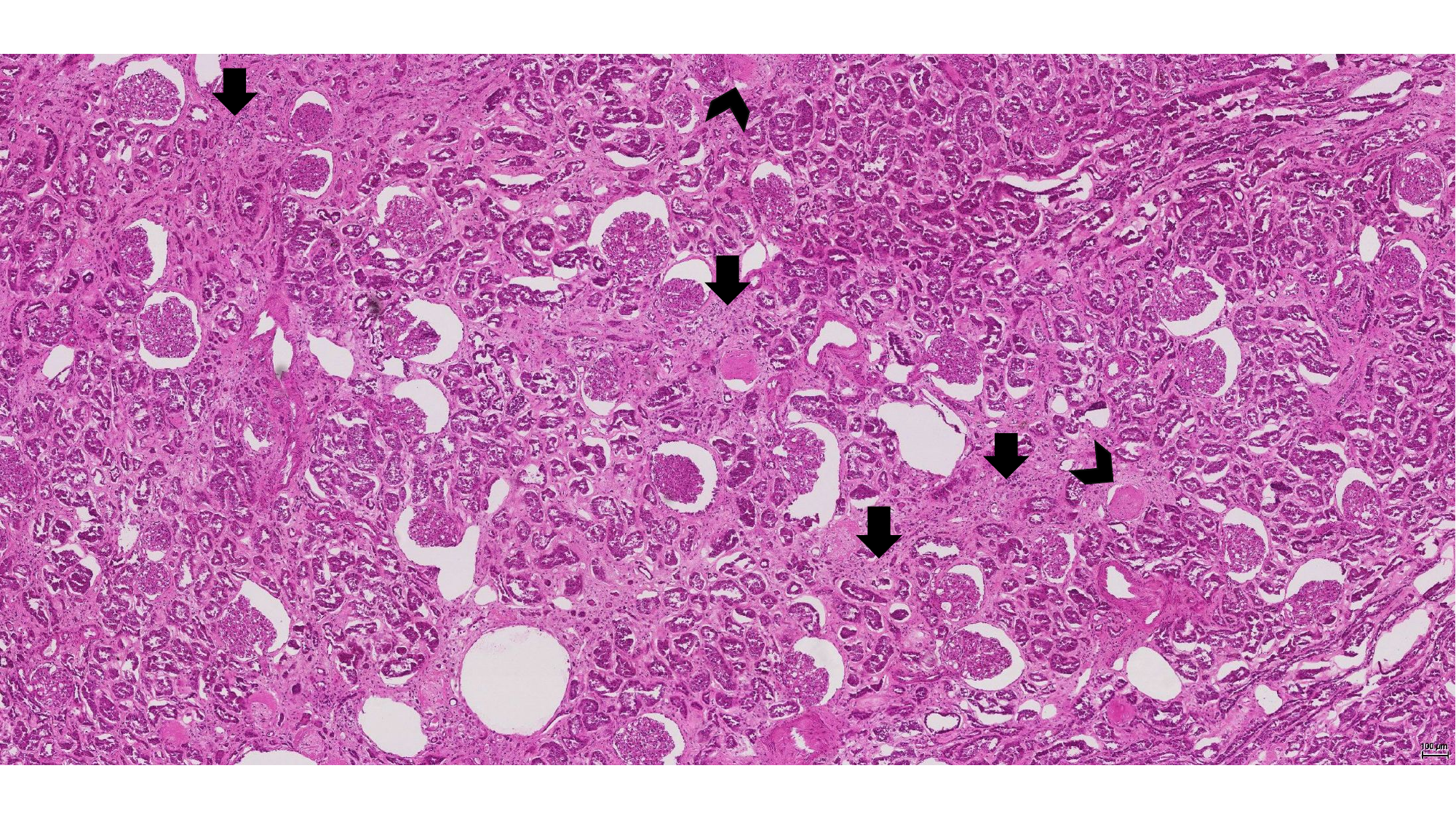

## Slide 5
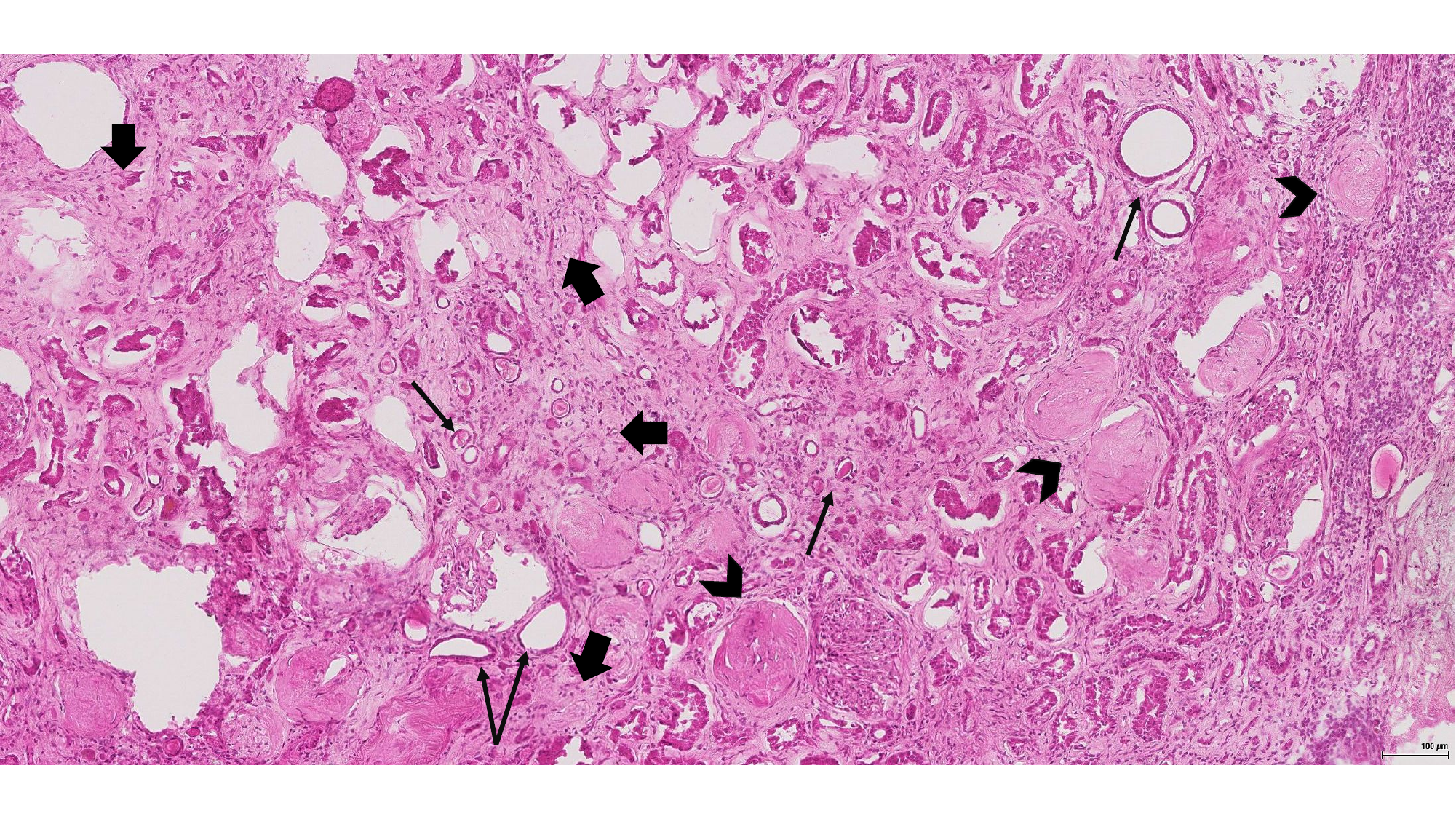

## Slide 6
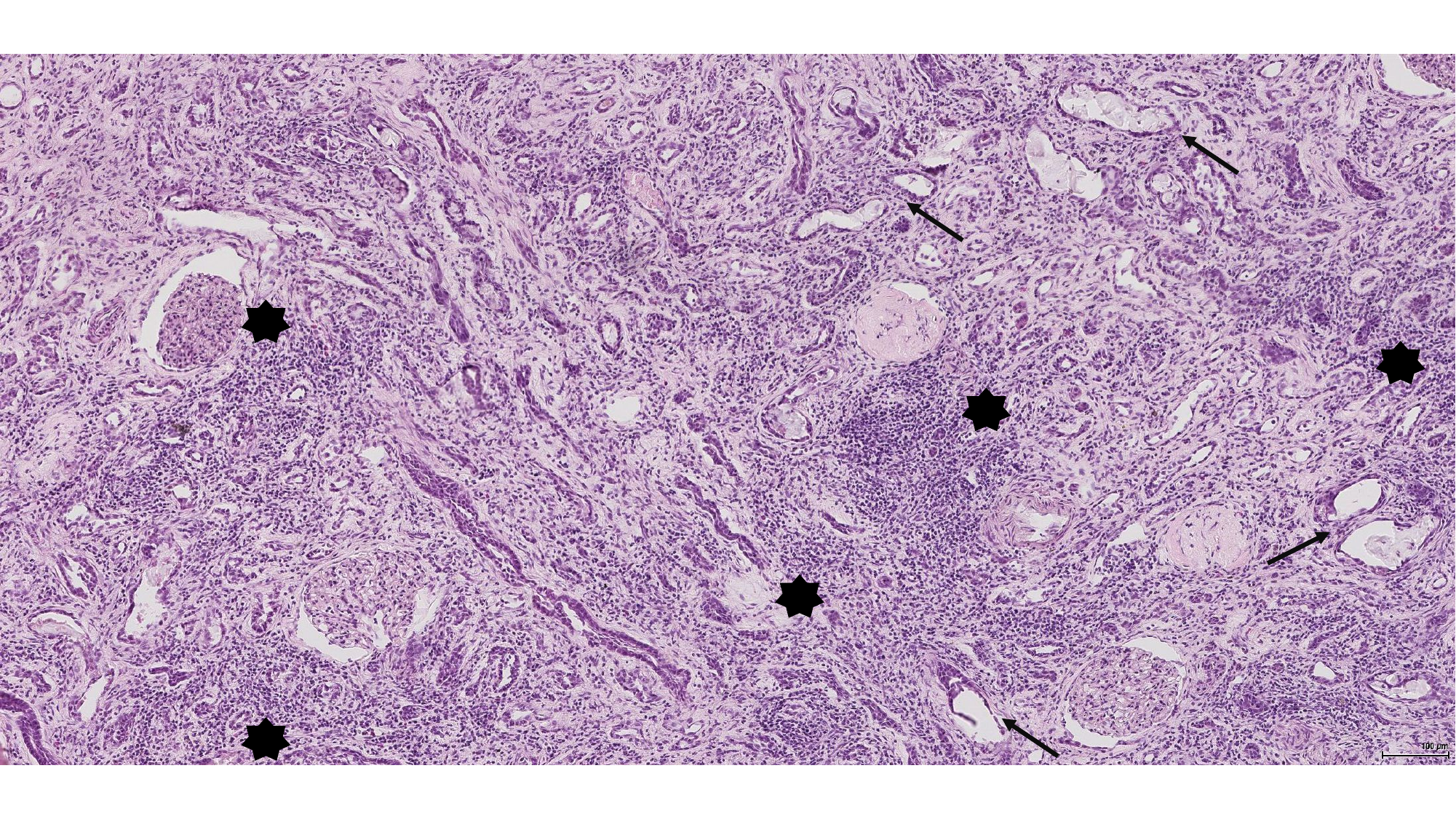

## Slide 7
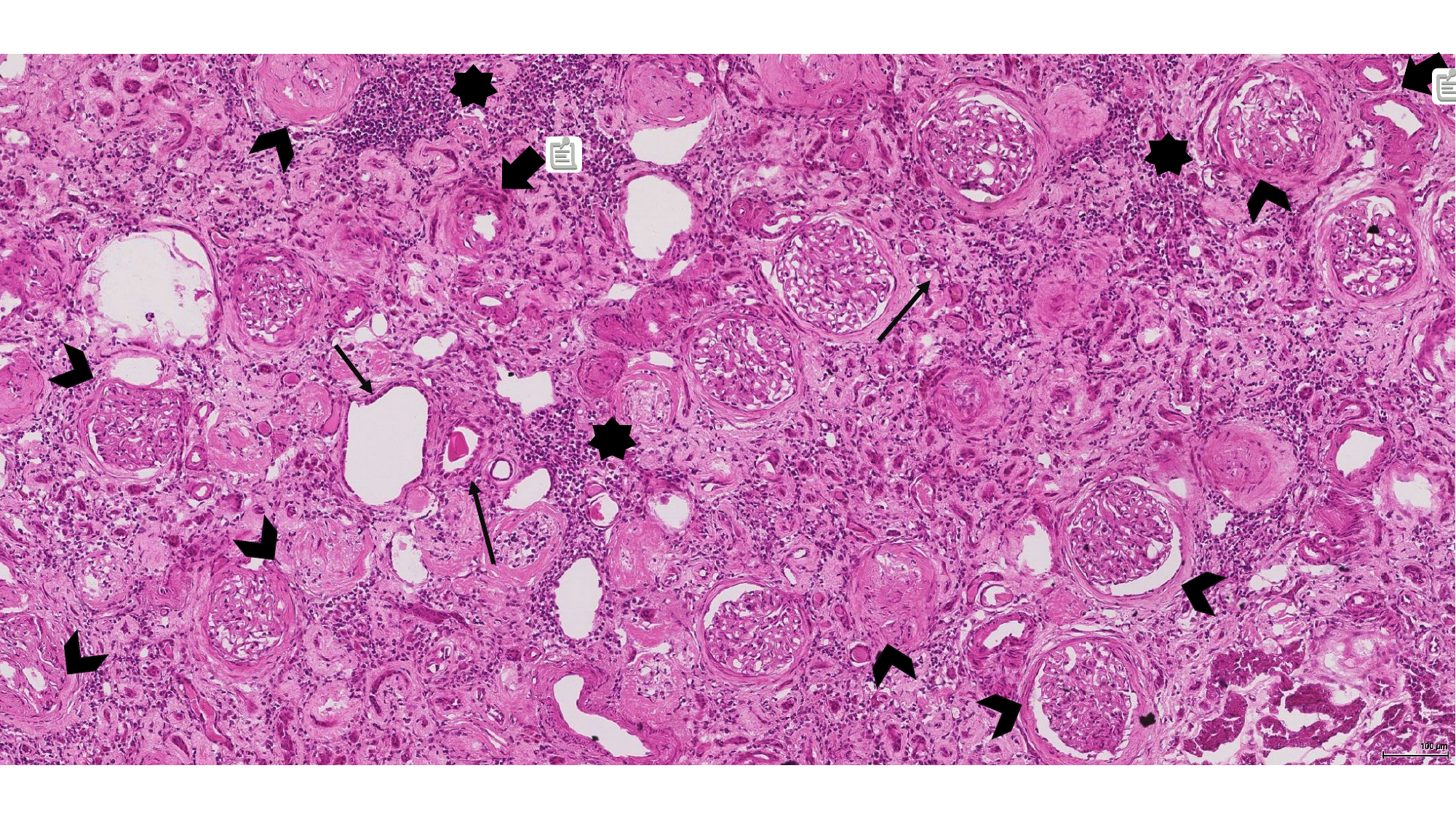

## Slide 8
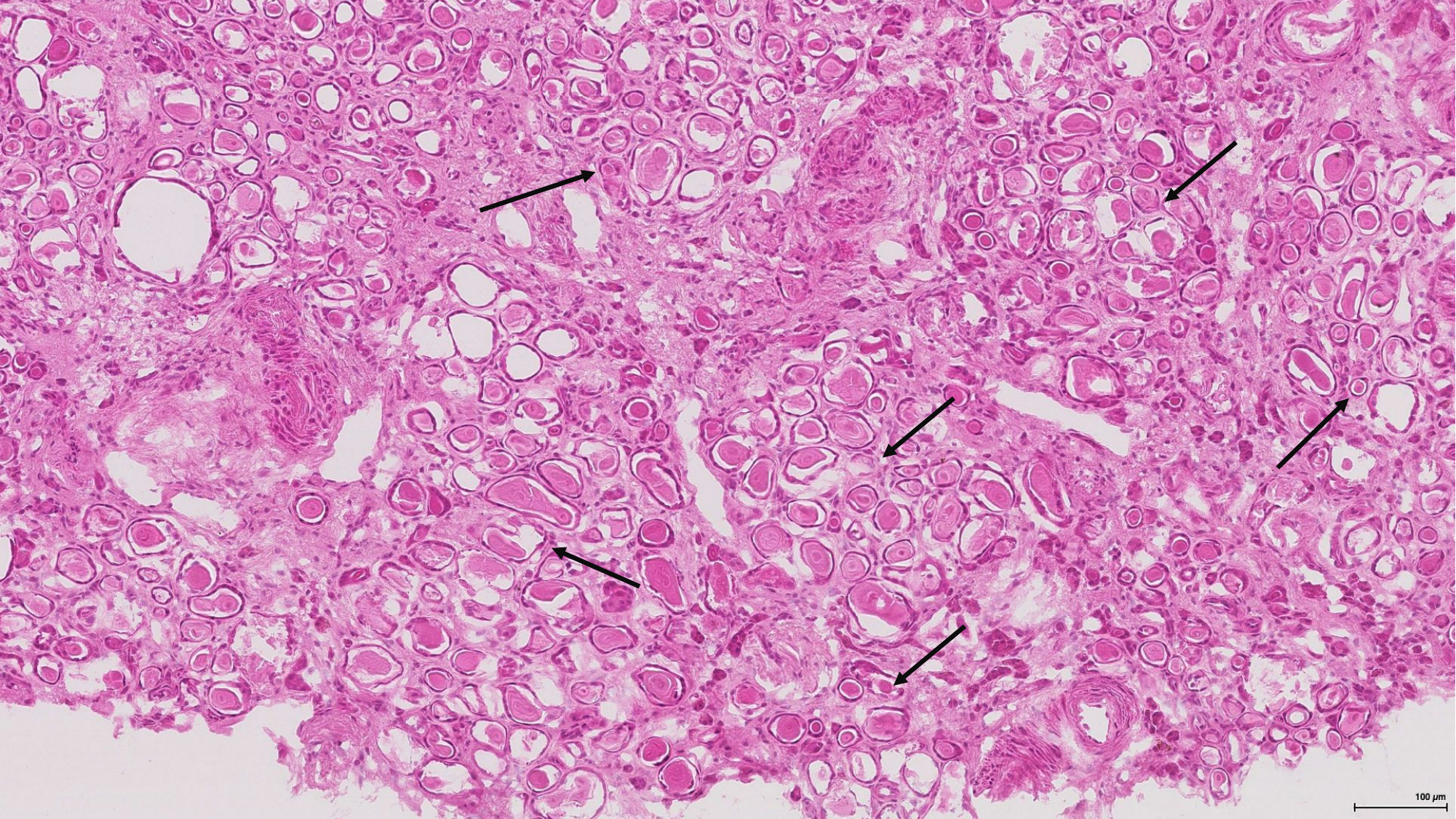

## Slide 9
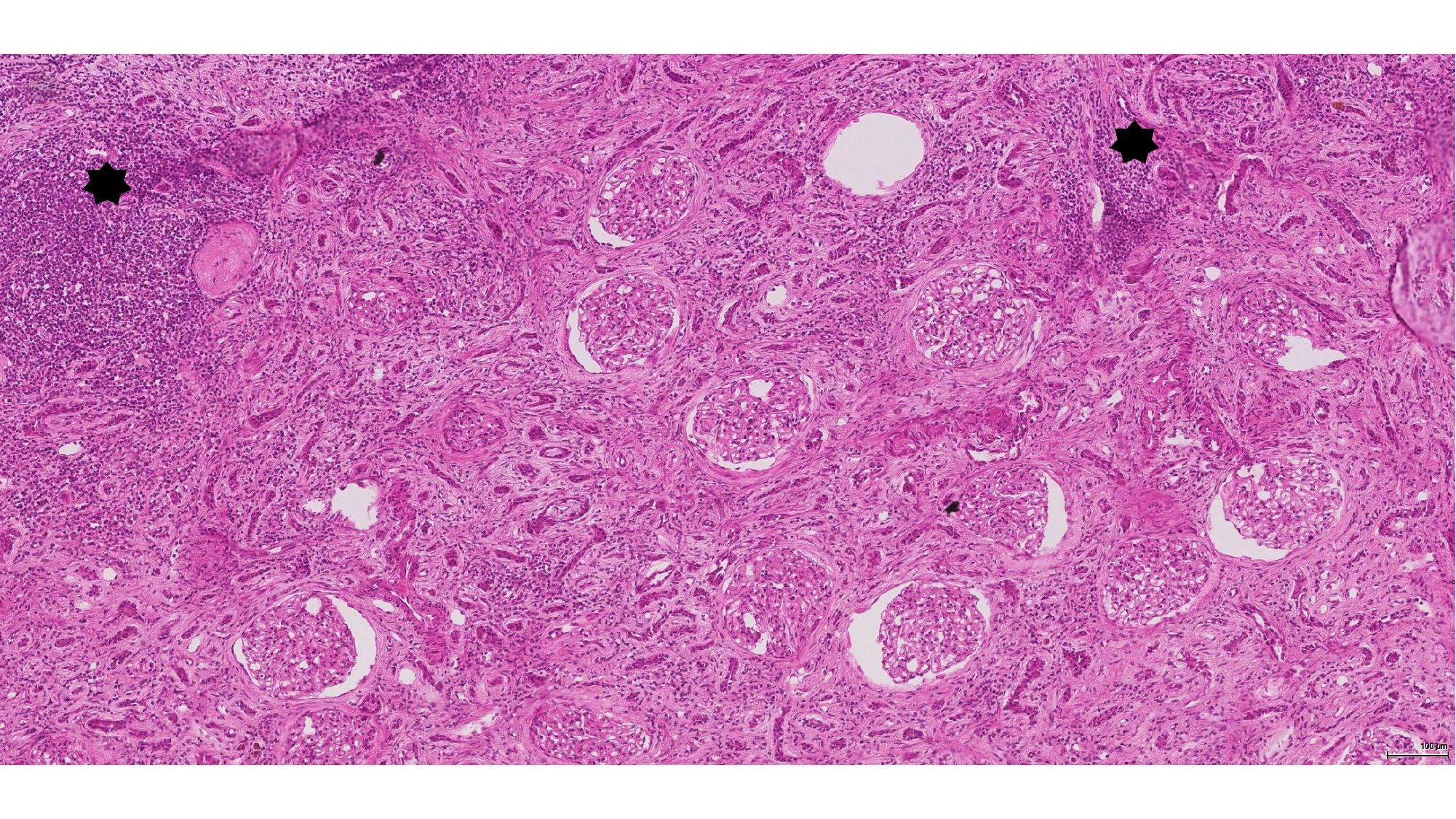

## Slide 10
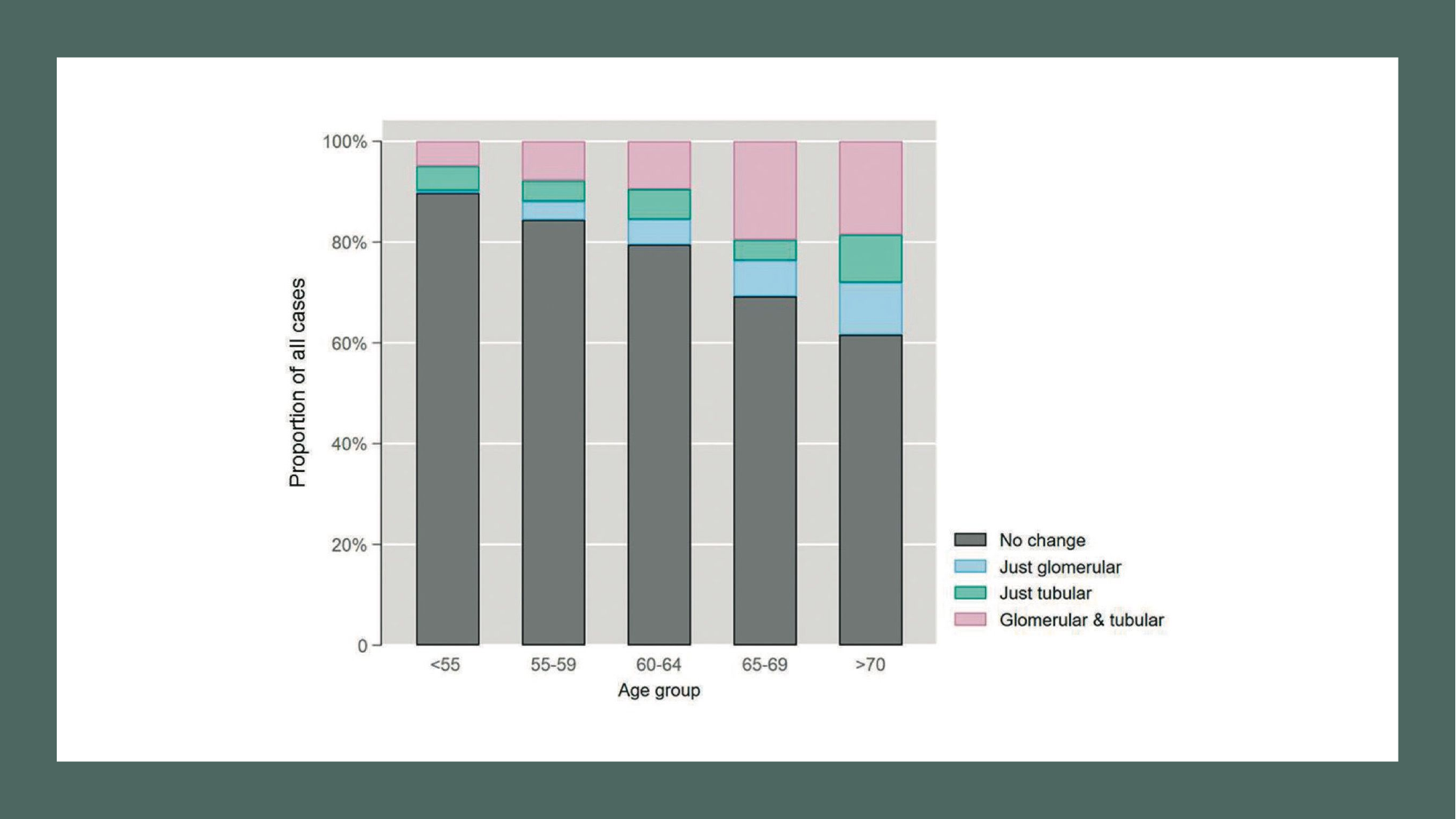

## Slide 11
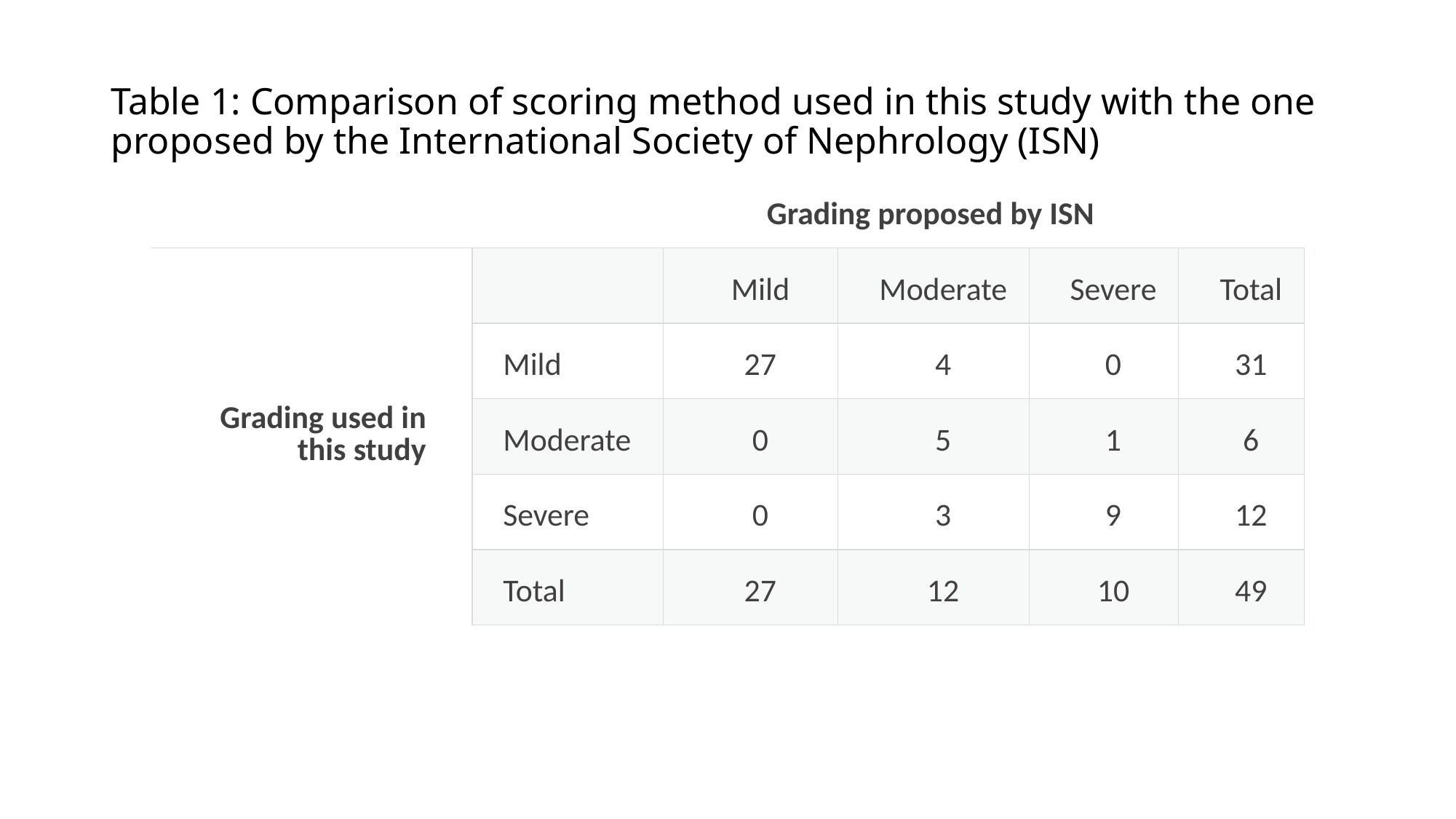

# Table 1: Comparison of scoring method used in this study with the one proposed by the International Society of Nephrology (ISN)
| | | Grading proposed by ISN | | | |
| --- | --- | --- | --- | --- | --- |
| | | Mild | Moderate | Severe | Total |
| Grading used in this study | Mild | 27 | 4 | 0 | 31 |
| | Moderate | 0 | 5 | 1 | 6 |
| | Severe | 0 | 3 | 9 | 12 |
| | Total | 27 | 12 | 10 | 49 |
