# Supplementary material for: Morphological findings in frozen non-neoplastic kidney tissues of patients with kidney cancer from large-scale multicentric studies on renal cancer
Source: Virchows Arch. 2021 Jan 5;478(6):1099–107. doi: 10.1007/s00428-020-02986-3 (PMC8203524; doi:10.1007/s00428-020-02986-3)
Supplement: Supplementary file 2 — (DOCX 15.1 kb) [file 428_2020_2986_MOESM2_ESM.docx]

**Supplementary Figures’ Legend:**

*Figures 1 & 2: none-mild group; Figures 3 to 7: moderate-severe group*

Figure 1: 61 Y/O patient from Romania, with history of both diabetes and hypertension, and no kidney disease. Scored as none (no significant pathological changes). Digital image, 10X

Figure 2: 68 Y/O patient from Czech Republic with history of hypertension, no diabetes or kidney disease. Some glomeruli are sclerotic (arrow heads). There is interstitial fibrosis and mild inflammation (thick arrows), scored as + (mild) CRPC. Digital image, 4X

Figure 3: 62 Y/O patient from Serbia, with history of hypertension only. Interstitial fibrosis (thick arrows) and tubular atrophy (thin arrows) are seen. Glomeruli are also affected (arrow heads), scored as ++ (moderate) CRPC. Digital image, 10X.

Figure 4: 45 Y/O patient from Czech Republic with history of hypertension only. Severe interstitial inflammation (star) is predominant with interstitial fibrosis. Some tubular atrophy (thin arrows) and a few numbers of sclerotic glomeruli are also seen, scored as ++ (moderate) CRPC. Digital image, 10X

Figure 5: 68 Y/O patient from Czech Republic with no history of diabetes, hypertension, or kidney disease. Parenchymal changes in all parenchymal elements is seen composed of variable levels of glomerular changes from basement membrane thickening to sclerosis (arrow heads), thick wall vessels (thick arrows), interstitial inflammation (star) and fibrosis, and tubular atrophy (thin arrows), scored as +++ (severe) CRPC. Digital image, 10X.

Figure 6: 40 Y/O patient from Serbia with no history of hypertension, diabetes, or kidney disease. There is severe and diffuse tubular atrophy filled with casts (thin arrows), leading to *thyroidization* pattern, in a background of interstitial fibrosis, scored as +++ (severe) CRPC. Digital image, 12X.

Figure 7: 71 Y/O patient from Romania with no medical history. Pathological findings are mainly as interstitial inflammation (star) and fibrosis. Some glomeruli are also fibrotic, scored as +++ (severe) CRPC. Digital image, 10X

Figure 8: Division of the tubular and glomerular chronic renal parenchymal changes by age categories.
